# Comparison of human immunodeficiency virus-related risky sexual behaviors between men who have sex with men only and men who have sex with men and women: A cross-sectional study in Eastern China

**DOI:** 10.1016/j.pmedr.2024.102923

**Published:** 2024-10-30

**Authors:** Rui Ge, Lin Chen, Wanjun Chen, Lin He, Chengliang Chai, Guoying Zhu, Zhongwen Chen

**Affiliations:** a Jiaxing Center for Disease Control and Prevention, Jiaxing, Zhejiang Province, China; b Department of HIV/AIDS and STDs Control and Prevention, Zhejiang Provincial Center for Disease Control and Prevention, Hangzhou, Zhejiang Province, China

**Keywords:** Human immunodeficiency virus, Men who have sex with men, Sexual behavior, Questionnaire, China

## Abstract

**Background:**

Men who have sex with men (MSM) are a significant population contributing to human immunodeficiency virus (HIV) transmission. Nevertheless, few studies have focused on the heterogeneity in MSM. The characteristics of men who have sex with men and women (MSMW) may differ from men who have sex with men only (MSMO).

**Methods:**

From October 2022 to March 2023, a cross-sectional study for MSM was conducted in Eastern China. An electronic questionnaire was used to collect the demographic characteristics and sexual behaviors of participants. A multivariable logistic regression model was used to assess the differences in HIV-related risky sexual behaviors between MSMO and MSMW.

**Results:**

A total of 1993 MSM were enrolled in the study, with 1221 MSMO and 772 MSMW. Compared to MSMW, MSMO reported younger age at first anal sex, higher coerced homosexual experiences, and observed different characteristics of sexual behavior in the past six months. Multivariable analysis indicated that MSMW were more likely to seek casual partners and use stimulants online and offline. MSMW showed inconsistent condom use with online partners (aOR = 0.51, 95 %CI: 0.38–0.68) and more frequent anal sex after drinking (aOR = 2.27, 95 %CI: 1.54–3.35). They also had higher risks of commercial (aOR = 2.47, 95 %CI: 1.71–3.57) and group sexual behaviors (aOR = 1.88, 95 %CI: 1.24–2.83) compared to MSMO.

**Conclusion:**

Our findings suggest that the differential factors may lead MSMW to become a subgroup at high risk of HIV infection. The gender identity and sexual orientation within MSM should be considered when formulating acquired immune deficiency syndrome (AIDS) prevention and control strategies.

## Highlights

• A study explored human immunodeficiency virus-related risky sexual behaviors.
• Data were sourced from 1993 men who have sex with men in Eastern China.
• The sexual behaviors of men who have sex with men subgroups are different.
• Bisexual behaviors are at high risk for human immunodeficiency virus infection.

## 1 Introduction

The implementation of comprehensive HIV prevention and control strategies has led to a decline in the incidence and mortality rates of HIV, as indicated by the data of Joint United Nations Programme on HIV/AIDS. However, the growing HIV/AIDS population has led to an increased HIV burden and poses challenges for AIDS prevention and control. Globally, an estimated 1.3 million individuals acquired HIV in 2022, with the total number of people living with HIV reaching 39 million (UNAIDS, 2023). Studies have revealed the low rate of condom use among MSM (Jin et al., 2013, Mann et al., 2022, Olawore et al., 2021) and the prevalent risky behaviors have increased the risk of HIV infection. MSM constitute a significant population affected by HIV, with a high prevalence of the disease observed within this group in many countries (Operario et al., 2022, Kloek et al., 2022). The estimated number of MSM was 8.3 million in mainland China (Hu et al., 2020).

MSMW constitute a crucial demographic group in the transmission of HIV across gender lines and represent a substantial reservoir for intrafamilial transmission, consequently heightening the risk of infection in women and facilitating mother-to-child transmission (Ito et al., 2021, Jeffries, 4th., 2014.). Certainly, there remains uncertainty and controversy regarding the HIV infection prevalence among bisexual individuals and the extent to which their behavior contributes to the spread of HIV among women. This further underscores the critical importance of conducting research on HIV-related sexual behaviors in MSMW (Ekstrand et al., 1994, O'Leary and Jones, 2006). Compared to MSMO, MSMW have indeed contributed to another public health concern in terms of heterosexual HIV transmission. This is because MSMW may engage in high-risk sexual behaviors with both male and female partners, which can increase the likelihood of HIV transmission to their female partners and subsequently to the general heterosexual population. Most studies have included MSMW only in samples of MSM, thereby masking the specific health issues faced by MSMW (Dodge et al., 2008). Furthermore, the results are inconsistent when comparing the prevalence of HIV among MSMW and MSMO (Martín-Sánchez et al., 2020, Bowring et al., 2016) The differences in demographic characteristics and HIV-related risky behaviors between MSMW and MSMWO vary across different periods, countries, and socio-cultural contexts (Jeffries and Johnson, 2018, Gaines et al., 2020, Hu et al., 2019). In China, the legal non-recognition of gay marriage and cultural pressures to “carry on the family name” lead many MSM to marry and have sexual relations with women. As a result, MSMW play a significant role as a conduit for HIV transmission, yet this issue has not been sufficiently addressed (Hu et al., 2019, Chen et al., 2015). In recent years, there has been an increase in heterosexual HIV infections in Zhejiang Province, Eastern China. Some of these cases may be linked to MSMW (Chen et al., 2023).

Previous studies have mainly focused on understanding the general characteristics and HIV/STD infection status of MSM and comparing them with other populations, with fewer studies exploring the internal differences within the MSM population (Martín-Sánchez et al., 2020, Friedman et al., 2014, Jeffries, 4th., 2014.). In contrast, we conducted a cross-sectional study in seven major cities in Zhejiang Province, with a specific emphasis on investigating the proportions and sexual behavior characteristics of MSMW and MSMO. Zhejiang Province, situated in eastern China, has a highly developed economy. In 2017, Zhejiang reported an HIV prevalence rate of 0.05 % with an estimated 28,520 people living with HIV (Chen et al., 2021). The prevalence of HIV among men who have sex with men (MSM) in Zhejiang has been 8 % since 2008. Additionally, more than 40 % of the newly diagnosed HIV-infected patients annually are MSM (He et al., 2024). Our study aimed to provide initial insights into the patterns of HIV-related risky behaviors among MSMW by comparing the differences in sexual behaviors between MSMW and MSMO. These findings would provide a basis for assessing the risk of transmission among MSM and developing personalized intervention strategies.

## 2 Methods

### 2.1 Study design and population

A cross-sectional study was conducted in the cities of Hangzhou, Ningbo, Jinhua, Wenzhou, Jiaxing, Shaoxing, and Quzhou in Zhejiang Province. MSM were recruited using the convenience sampling method by Non-Governmental Organizations (NGOs) through various channels, including online testing platforms, dating apps (such as WeChat, QQ, and Blued), and offline venues. In this study, we classified MSM into MSMO and MSMW according to their self-reported sexual practices. MSMO were defined as participants who reported having only male sexual partners prior to the survey. Meanwhile, MSMW were defined as participants who had at least one male and one female partner. We recruited eligible subjects according to the following criteria. Inclusion criteria: (1) Participants aged ≥ 18 years old, who were born male and identified as male at the time of the survey, and those who self-reported having ever engaged in oral or anal sex with a man. (2) Participants who consented to an HIV test were given either a standard blood test or a rapid test, followed by laboratory confirmation according to local procedures. Participants with negative HIV antibody test results were included in the study to avoid those with positive HIV antibody test results refusing to answer or concealing sexual behavior-related information. (3) consent and voluntary participation in this study. Exclusion criteria: those who were unable to complete the informed consent due to intellectual or mental factors.

This study was approved by the Ethics Committee of the Zhejiang Center for Disease Control and Prevention with the serial number 2022–009-01. All data used in this study followed the Law of the Prevention and Treatment of Infectious Diseases in China and the Declaration of Helsinki. All study participants were provided with informed consent and signed an electronic consent form on their mobile phones at the time of enrollment.

### 2.2 Questionnaire

Questionnaires were distributed by the NGOs in conjunction with their testing work platforms, and electronic questionnaires were distributed on these platforms. Upon completion of participant recruitment, individuals signed the electronic informed consent form on-site under the guidance of trained volunteers, and those who consented were directed to the website to complete the questionnaire. The questionnaire developed by the National Center for AIDS/STD Control and Prevention included self-reported information on demographic characteristics, psychosocial situation, AIDS knowledge, sexual behaviors, and HIV testing status. Demographic information, smoking history, drinking history, hypertension history, and general medical history were collected for all participants using a combined questionnaire.

### 2.3 Measurement

#### 2.3.1 Demographic characteristics

The demographic information collected in this study includes the following aspects: age; registration (Zhejiang province or others); type of residence (urban or rural); education (middle school and under, high school or equivalent, or college and above); income (≤90,000 Chinese Yuan per year or > 90,000 Chinese Yuan per year); marital status (unmarried, divorced/cohabitation, or married/cohabitation); sexual orientation (homosexual, bisexual, or heterosexual/unsure); sex role (most insertive, most receptive, or both).

#### 2.3.2 Sexual behaviors

Among the study participants, self-reported sexual behavior information was collected through questionnaires to understand the patterns and risks associated with their sexual behaviors. The questionnaire-collected sexual behavior information of participants included the age at which they had their first anal sex with a man. Additionally, participants were asked to answer “Yes/No” to the following questions regarding their sexual behaviors in the past six months: For online sexual behaviors, have you sought online casual sex partners? If so, have you consumed stimulants (such as Rush Popper) with online casual sex partners? Have you used condoms with online casual sex partners? For offline sexual behaviors, have you sought offline casual sex partners? If so, have you consumed stimulants (such as Rush Popper) with offline casual sex partners? Have you used condoms with offline casual sex partners? And for other risky behaviors, was there anal sex after drinking? Was there commercial sex? Was there group sex? Was there anal sex after using new psychoactive substances? To ensure the authenticity and confidentiality of participants' responses, strict data collection procedures were followed. All reactions were anonymized and handled with the utmost care to protect the privacy of the respondents.

### 2.4 Statistical analysis

A descriptive analysis was performed to outline the general epidemiological characteristics of the demographic data. Quantitative variables were reported as mean ± SD, while qualitative variables were presented as counts and proportions. T-tests were utilized for continuous variables, while chi-square tests were employed for categorical variables. The chi-square tests were utilized to examine MSM in terms of demographic characteristics and sexual behaviors. The comparison of HIV-related risky sexual behaviors between MSMW and MSMO was evaluated using a multivariable logistic regression model. MSMW and MSMO served as dependent variables, with MSMO being the reference. For the selected independent variables, we evaluated their association with MSMW after adjusting for potential confounders such as age, registration, type of residence, education, income, and marital status. The regression model results consist of adjusted odds ratios (aORs), and 95 % confidence intervals (CIs). SPSS 26.0 software was utilized for the statistical analysis. A *P*-value less than 0.05 was considered statistically significant.

## 3 Results

### 3.1 Demographic characteristics

**Fig. 1:**
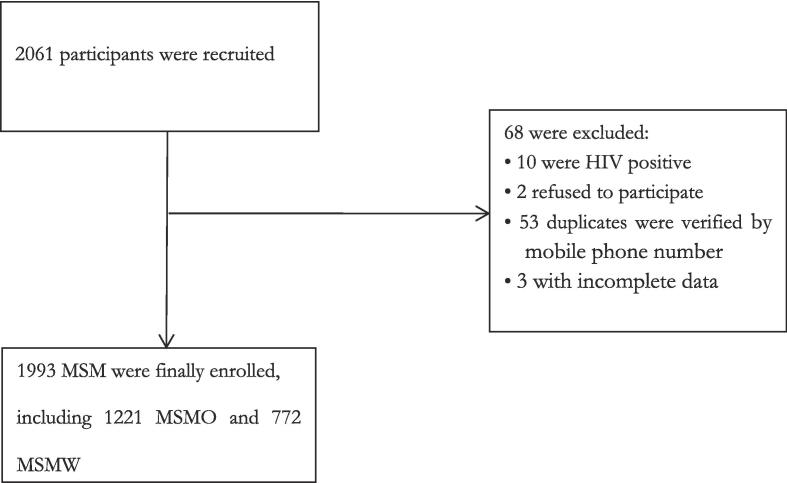
Flow chart of participants’ enrolment in the cross-sectional study between October 2022 and March 2023. Abbreviations: MSM, men who have sex with men; MSMO, men who have sex with men only; MSMW, men who have sex with men and women; HIV, human immunodeficiency virus.

During the period from October 2022 to March 2023, a total of 2061 individuals were initially recruited in the seven cities of Hangzhou, Ningbo, Jinhua, Wenzhou, Jiaxing, Shaoxing, and Quzhou. Among them, 68 MSM were excluded from the study due to not meeting the inclusion criteria. Consequently, 1993 MSM were ultimately enrolled, comprising 1221 (61.3 %) MSMO and 772 (38.7 %) MSMW. The participant enrollment process is illustrated in Fig. 1.

The mean age of MSMO was 37.25 ± 9.06 years, and that of MSMW was 27.56 ± 7.17 years. The percentage of individuals with a college education or higher was greater among MSMO compared to MSMW (67.9 % vs 43.7 %, *P* < 0.001). Additionally, the proportion of individuals in the income group of ≤ 90,000 was higher in MSMO than in MSMW (68.0 % vs 63.1 %, *P* = 0.025). The percentage of unmarried individuals was significantly higher among MSMO compared to MSMW (89.3 % vs 27.1 %, *P* < 0.001). Moreover, the proportion of individuals identifying as homosexual was greater in MSMO than in MSMW (74.2 % vs 38.5 %, *P* < 0.001). Conversely, the percentage of individuals reporting the “most insertive role” was lower in MSMO than in MSMW (40.7 % vs 53.8 %, *P* < 0.001). There were no significant differences in registration status and type of residence between MSMO and MSMW (*P* > 0.05). The detailed results are provided in Table 1.

**Table 1:** Demographic characteristics of men who have sex with men only and men who have sex with men and women in our study in our study between October 2022 and March 2023, *N* = 1993.

Variable	MSMO (n = 1221)	MSMW (n = 772)	*P-*value
Registration			
Zhejiang province	760(62.2)	463(60.0)	0.311
other provinces	461(37.8)	309(40.0)	
Type of residence			
Urban	957(78.4)	616(79.8)	0.451
Rural	264(21.6)	156(20.2)	
Education			
Middle school and under	132(10.8)	189(24.5)	0.000
High school or equivalent	260(21.3)	246/(31.9)	
College and above	829(67.9)	337(43.7)	
Income (CNY/year)			
≦ 90,000	830(68.0)	487(63.1)	0.025
> 9,0000	391(32.0)	285(36.9)	
Marital status			
Unmarried	1090(89.3)	209(27.1)	0.000
Divorced/cohabitation	31(2.5)	112(14.5)	
Married/cohabitation	100(8.2)	451(58.4)	
Sexual orientation			
homosexual	906(74.2)	297(38.5)	0.000
bisexual	210(17.2)	387(50.1)	
heterosexual/unsure	105(8.6)	88(11.4)	
Sex role			
Most insertive	497(40.7)	415(53.8)	0.000
Most receptive	305(25.0)	180(23.3)	
Both	419(34.3)	177(22.9)	

Note: Data presented as n (%). The chi-squared tests was used to compare the differences of demographic characteristics between MSMO and MSMW. *P*-values obtained from chi-square statistics less than 0.05 was considered statistically significant.

CNY, Chinese Yuan; MSMO, men who have sex with men only; MSMW, men who have sex with men and women.

### 3.2 Sexual behaviors

The study found significant differences between MSMO and MSMW in various aspects related to their sexual behaviors: (1) Age of First Anal Sex with a man: The mean age of first anal sex with a man was younger in MSMO compared to MSMW (21.23 ± 4.72 vs 26.18 ± 7.68, *P* < 0.001). (2) Circumstances of First Homosexual Experience: A higher percentage of MSMO were coerced, induced, or lured with money for their first homosexual experience compared to MSMW (76.0 % vs 69.4 %, *P* = 0.001). (3) Sexual behaviors in the Past six Months: Significant differences were observed between MSMO and MSMW in various sexual behaviors in the past six months, including: Finding online casual sex partners (*P* < 0.001); Stimulant use with online casual sex partners (*P* = 0.002); Condom use with online casual sex partners (*P* = 0.002); Finding offline casual sex partners (*P* < 0.001); Stimulant use with offline casual sex partners (*P* = 0.009); Condom use with offline casual sex partners (*P* < 0.001); Engaging in anal sex after drinking (*P* < 0.001); Involvement in commercial sexual behavior (*P* < 0.001); Participation in group sexual behavior (*P* = 0.002). The results are presented in Table 2.

**Table 2:** The comparison of sexual behaviors between men who have sex with men only and men who have sex with men and women in our study between October 2022 and March 2023, *N* = 1993.

Variable	MSMO (n = 1221)	MSMW (n = 772)	*P-*value
Age of first anal sex with a man (year)	21.23 ± 4.72	26.18 ± 7.68	0.000
Circumstances of first homosexual experience			
Proactively and voluntarily	293(24.0)	236(30.6)	0.001
Coerced/induced/money lured	928(76.0)	536(69.4)	
Finding online casual sex partners in the past six months			
No	539(44.1)	268(34.7)	0.000
Yes	682(55.9)	504(65.3)	
Stimulant use with online casual sex partners in the past six months			
No	430(63.1)	273(54.2)	0.002
Yes	252(37.0)	231(45.8)	
Condom use with online casual sex partners in the past six months			
Consistent	461(67.6)	297(58.9)	0.002
Inconsistent	221(32.4)	207(41.1)	
Finding offline casual sexual partners in the past six months			
No	950(77.8)	507(65.7)	0.000
Yes	271(22.2)	265(34.3)	
Stimulant use with offline casual sex partners in the past six months			
No	143(52.8)	110(41.5)	0.009
Yes	128(47.2)	155(58.5)	
Condom use with offline casual sex partners in the past six months			
inconsistent	1878(69.0)	135(50.9)	0.000
consistent	84(31.0)	130(49.1)	
Anal sex after drinking in the past six months			
No	1150(94.2)	692(89.6)	0.000
Yes	71(5.8)	80(10.4)	
Commercial sexual behavior in the past six months			
No	1170(95.8)	695(90.0)	0.000
Yes	51(4.2)	77(10.0)	
Group sexual behavior in the past six months			
No	1176(96.3)	720(93.3)	0.002
Yes	45(3.7)	52(6.7)	
Anal sex after New psychoactive substances use in the past six months			
No	1214(99.4)	770(99.7)	0.308
Yes	7(0.6)	2(0.3)	

Note: Data presented as the mean ± SD or n (%). Statistical significance of differences between groups was evaluated by employing T-tests for continuous variables and Chi-squared tests for categorical variables. *P*-values obtained from chi-square statistics less than 0.05 was considered statistically significant.

MSMO, men who have sex with men only; MSMW, men who have sex with men and women.

### 3.3 Comparison of HIV-related risky sexual behaviors among MSMW and MSMO

The results of a multivariable logistic regression analysis revealed significant differences between MSMW and MSMO in the prevalence of HIV-related risky sexual behaviors in the past six months, even after controlling for variables such as age, registration status, type of residence, education level, income, and marital status. MSMW exhibited a higher likelihood of seeking casual partners online (aOR = 1.49, 95 %CI: 1.20–1.86) and engaging in stimulant use with online casual partners (aOR = 1.58, 95 %CI: 1.16–2.15) compared to MSMO. Additionally, MSMW demonstrated an increased risk of encountering offline casual sexual partners (aOR = 1.67, 95 %CI: 1.37–2.06) and engaging in stimulant use with offline casual partners (aOR = 1.82, 95 %CI: 2.28–2.62). Notably, MSMW were less inclined to use condoms with online casual partners (aOR = 0.51, 95 %CI: 0.38–0.68), while they exhibited a higher rate of condom use with offline casual partners in comparison to MSMO (aOR = 2.50, 95 %CI: 1.70–3.67). The odds of engaging in anal sex after drinking were 2.27 times higher among MSMW than among MSMO (aOR = 2.27, 95 %CI: 1.54–3.35). Furthermore, MSMW showed an increased risk of commercial sexual behavior (aOR = 2.47, 95 %CI: 1.71–3.57) and group sexual behavior compared to MSMO (aOR = 1.88, 95 %CI: 1.24–2.83). See Table 3.

**Table 3:** Multivariable logistic regression results with human immunodeficiency virus-related risky sexual behaviors in the past six months as dependent variable in our study between October 2022 and March 2023, *N* = 1993.

Variable	aOR	95 % CI
Finding online casual sex partners in the past six months		
MSMW	1.49	1.20–1.86
MSWO	1.00	
Stimulant use with online casual sex partners in the past six months		
MSMW	1.58	1.16–2.15
MSWO	1.00	
Condom use with online casual sex partners in the past six months		
MSMW	0.51	0.38–0.68
MSWO	1.00	
Finding offline casual sexual partners in the past six months		
MSMW	1.67	1.36–2.06
MSWO	1.00	
Stimulant use with offline casual sex partners in the past six months		
MSMW	1.83	2.28–2.62
MSWO	1.00	
Condom use with offline casual sex partners in the past six months		
MSMW	2.50	1.70–3.67
MSWO	1.00	
Anal sex after drinking in the past six months		
MSMW	2.27	1.54–3.35
MSWO	1.00	
Commercial sexual behavior in the past six months		
MSMW	2.47	1.71–3.57
MSWO	1.00	
Group sexual behavior in the past six months		
MSMW	1.88	1.24–2.83
MSWO	1.00	

Note: The model controlling for age, registration, education, income and marital status.

MSMO, men who have sex with men only; MSMW, men who have sex with men and women; aOR = adjusted odds ratio; 95 % CI = 95 % confidence interval.

## 4 Discussion

This study was based on a cross-sectional investigation conducted in Zhejiang province, located in the eastern part of China. The region features a developed economy, a dense population, and a lively MSM community. Conducting research in this area would enhance our understanding of the characteristics of the MSM population in China. Our study revealed that 38.7 % of MSM exhibited bisexual behavior. This finding was consistent with similar studies conducted in the United States and India (Mc et al., 2017, Ramakrishnan et al., 2015, Brahmam et al., 2008) but was higher than the 16.1 % reported in Western China (Hu et al., 2019) and the 13.4 % observed in Melbourne, Australia (Martín et al., 2020). This disparity calls for a more in-depth examination of the regional and cultural variations in sexual behaviors. For instance, an investigation in the United States could offer valuable insights into how cultural, social, and economic factors shape the MSM subgroups’ sexual behaviors and HIV risk (Dyer et al., 2015).

In comparing MSMW and MSMO, distinct differences in demographic characteristics and sexual behaviors were observed. MSMW tend to be older, less educated, have higher incomes, and have a greater proportion of bisexual individuals compared to MSMO. Chayada, et al in Thailand reported similar trends in the analysis of MSM subgroups (Chaiyabutr et al., 2022). Additionally, MSMW exhibited a higher rate of being in a marital relationship and a preference for the insertive role during anal sex (Gaines et al., 2020, Parchem et al., 2022). Previous studies demonstrated that the risk of HIV exposure varies across sex roles. The risk of HIV infection was higher for individuals in the receptive role compared to those in the insertive role (Stansfield et al., 2019, Goodreau et al., 2005). Therefore, both male and female partners of MSMW are at risk of HIV transmission from them (Baggaley et al., 2010).

Regarding sexual behaviors, MSMO exhibited a lower mean age at the onset of homosexual behavior and a higher percentage of involuntary participation. The mean age of first anal sex among MSMO was 21.23 ± 4.721 years, and the rate of involuntary participation was 76 %. This was consistent with the global pattern indicating a higher HIV prevalence among MSM aged 15–24 years (UNAIDS, 2023, Shannon and Klausner, 2018, Zhao et al., 2020). A previous study pointed out that children and adolescents among MSM are closely associated with sexual violence (Kirwan et al., 2023). Our study highlights the importance of paying particular attention to the sexual psychology of adolescents and young adults, and intimate partner violence when conducting HIV prevention interventions among MSM (Miltz et al., 2019). Furthermore, the differences in sexual behaviors in the past six months between MSMO and MSMW were significant. After adjusting for variables such as age, household registration status, residential type, education level, income, and marital status, we compared MSMW and MSMO engaged in various HIV-related risky behaviors to identify differences between them. Compared to MSMO, MSMW are more likely to seek temporary partners online and offline, engage in commercial sex, and participate in group sex. In comparison to traditional offline socializing methods, online dating offers greater convenience and selectivity and is favored by MSM. Many MSM showed a high frequency of using dating websites and apps (Badal et al., 2018). A study on MSM dating practices in the United States revealed that 71.8 % of respondents used online tools to seek partners (Pravosud et al., 2022). The interaction between HIV stigma and the anonymity of online dating results in online dating having a greater degree of unpredictability and a higher risk of HIV infection compared to offline dating (Heijman et al., 2016). Providing HIV testing and pre-exposure prevention services through the Internet would benefit MSM (Cheah et al., 2024, Zhou et al., 2023). Certainly, online HIV prevention and control campaigns could be tailored according to the unique characteristics of MSMW and MSMO.

The authors also found that MSMW were more likely to use stimulants with online and offline casual sex partners compared with MSMO. Stimulant use is a widespread and challenging problem among MSM, serving as a significant driver of HIV transmission through multiple sexual partners and the practice of condomless anal sex (Mimiaga et al., 2018, Rosińska et al., 2018). The co-occurrence of stimulant use and risky sexual behavior among MSMW may make them a subgroup of MSM at high risk of HIV infection. In terms of condom use, MSMW had a lower rate of use than MSMO in the past six months. Moreover, MSMW showed differences in condom use with online and offline partners. Researches suggest that compared to offline partners, MSMW are more likely to engage in sexual behaviors with online partners without using condoms, which is consistent with our findings (Nyoni and James, 2022, Davis et al., 2016). This may be due to the anonymity and distance between individuals during online interactions, which could reduce awareness and vigilance towards sexual health risks. Our study indicates that it is advisable to always use condoms during sexual activities, regardless of whether they involve online or offline partners, to protect oneself and others from the risk of HIV infection. Future research should strive to broaden the scope of comparison and explore potential interventions based on these disparities.

Our findings should be considered in light of the following limitations. Firstly, the participants in the study were a convenience sample. This may lead to selection bias as the samples might not represent the target population, resulting in overestimation or underestimation of characteristics or outcomes. It can also cause response bias since the participants may have certain characteristics that affect their participation. Moreover, non-randomness can cause sampling errors. Secondly, the questionnaires involved inquiries about past sexual behaviors, potentially leading to information bias and recall bias. Finally, being a cross-sectional study means that causal relationships cannot be established. It is recommended that our findings be verified in a cohort study.

## 5 Conclusion

In summary, this study found that there were significant differences in demographic characteristics and sexual behaviors between MSMW and MSMO. Our findings suggest that the differential factors of MSMW may lead them to become a subgroup at high risk of HIV infection in the MSM population, including more online dating, stimulant use, inconsistent condom use, and commercial and group sexual activities. Therefore, gender identity and sexual orientation within MSM should be considered when formulating AIDS prevention and control strategies. Our study provides valuable perspectives on the characteristics and sexual behaviors of MSMW and MSMO in Eastern China. However, further research is essential to better comprehend the regional and global variations in these behaviors and develop more effective HIV prevention strategies.

## CRediT authorship contribution statement

**Rui Ge:** Writing – original draft, Methodology, Investigation, Funding acquisition. **Lin Chen:** Writing – review & editing, Supervision, Funding acquisition. **Wanjun Chen:** Writing – review & editing, Resources, Investigation. **Lin He:** Writing – review & editing, Supervision, Funding acquisition. **Chengliang Chai:** Writing – review & editing, Supervision, Resources. **Guoying Zhu:** Writing – review & editing, Supervision, Investigation, Data curation. **Zhongwen Chen:** Writing – review & editing, Supervision, Conceptualization.

## Funding

This study was supported by the 10.13039/501100017594Medical Science and Technology Project of Zhejiang Province, China (Grant No. 2021RC048), Zhejiang Provincial Program for the Cultivation of High-Level Innovative Health Talents (Grant No. 2021-132), Zhejiang Science and Technology Plan for Disease Prevention and Control (Grant No. 202548048 and 202550856), and “Innovative Jiaxing Talent support program” Top Health Talents (Grant No. 2069901).

## Declaration of competing interest

The authors declare that they have no known competing financial interests or personal relationships that could have appeared to influence the work reported in this paper.

## Data Availability

Data will be made available on request.

## References

[b0005] Badal H.J., Stryker J.E., DeLuca N., Purcell D.W. (2018). Swipe Right: Dating Website and App Use Among Men Who Have Sex With Men. AIDS. Behav..

[b0010] Baggaley R.F., White R.G., Boily M.C. (2010). HIV transmission risk through anal intercourse: systematic review, meta-analysis and implications for HIV prevention. Int. J. Epidemiol..

[b0015] Bowring A.L., Veronese V., Doyle J.S., Stoove M., Hellard M. (2016). HIV and sexual risk among men who have sex with men and women in Asia: A systematic review and meta-analysis. AIDS. Behav..

[b0020] Brahmam G.N., Kodavalla V., Rajkumar H. (2008). Sexual practices, HIV and sexually transmitted infections among self-identified men who have sex with men in four high HIV prevalence states of India. AIDS..

[b0025] Chaiyabutr C., Nanchaipruek Y., Pochanapan O., Leeyaphan C., Jiamton S. (2022). Characteristics of HIV/sexually transmitted infections among men who have sex with men and women in Bangkok. Thailand. Int. J. STD. AIDS..

[b0030] Cheah M.H., Gan Y.N., Altice F.L. (2024). Testing the Feasibility and Acceptability of Using an Artificial Intelligence Chatbot to Promote HIV Testing and Pre-Exposure Prophylaxis in Malaysia: Mixed Methods Study. JMIR. Hum. Factors..

[b0035] Chen J.P., Han M.M., Liao Z.J. (2015). HIV-related behaviors, social support and health-related quality of life among men who have sex with men and women (MSMW): a cross-sectional study in Chongqing. China. Plos One..

[b0040] Chen L., Luo M., Xu Y. (2021). The first 90: Progress in HIV detection in Zhejiang Province, 2008–2018. PLoS One..

[b0045] Chen L., Jiang T., Wang H. (2023). Development and validation of a risk score for predicting inconsistent condom use with women among men who have sex with men and women. BMC Public Health..

[b0050] Davis A., Best J., Luo J. (2016). Differences in risk behaviours, HIV/STI testing and HIV/STI prevalence between men who have sex with men and men who have sex with both men and women in China. Int. J. STD. AIDS..

[b0055] Dodge B., Jeffries W.L., Sandfort T.G. (2008). Beyond the Down Low: sexual risk, protection, and disclosure among at-risk Black men who have sex with both men and women (MSMW). Arch. Sex. Behav..

[b0060] Dyer T.P., Regan R., Pacek L.R., Acheampong A., Khan M.R. (2015). Psychosocial vulnerability and HIV-related sexual risk among men who have sex with men and women in the United States. Arch. Sex. Behav..

[b0065] Ekstrand, ML., Coates, TJ., Guydish, JR., Hauck, WW., Collette, L., Hulley, SB., 1994. Are bisexually identified men in San Francisco a common vector for spreading HIV infection to women? Am. J. Public Health. Jun;84(6):915-9. doi: 10.2105/ajph.84.6.915. 10.2105/ajph.84.6.915 PMC1614964 8203686

[b0070] Friedman M.R., Stall R., Silvestre A.J. (2014). Stuck in the middle: longitudinal HIV-related health disparities among men who have sex with men and women. J. Acquir. Immune. Defic. Syndr..

[b0075] Gaines, MT., McCree, DH., Gaul, Z., Henny, KD., Hickson, DA., Sutton, MY., 2020. Comparison of selected sociodemographic characteristics and sexual risk behaviors of black/african american men who have sex with men only and men who have sex with men and women, southeastern United States, 2013–2016. J. Racial. Ethn. Health, Disparities. 7(1):84–89. doi: 10.1007/s40615-019-00636-2. 10.1007/s40615-019-00636-2 PMC6980258 31502106

[b0080] Goodreau, SM., Goicochea, LP., Sanchez J., 2005. Sexual role and transmission of HIV Type 1 among men who have sex with men, in Peru. J. Infect. Dis. 191 Suppl 1(0 1):S147-58. doi: 10.1086/425268. 10.1086/425268 PMC4063354 15627225

[b0085] He L., Jiang T., Chen W. (2024). Examining HIV Testing Coverage and Factors Influencing First-Time Testing Among Men Who Have Sex With Men in Zhejiang Province, China: Cross-Sectional Study Based on a Large Internet Survey. JMIR. Public Health. Surveill..

[b0090] Heijman T., Stolte I., Geskus R. (2016). Does online dating lead to higher sexual risk behaviour? A cross-sectional study among MSM in Amsterdam, the Netherlands. BMC Infect Dis..

[b0095] Hu M., Xu C., Wang J. (2020). Spatiotemporal Analysis of Men Who Have Sex With Men in Mainland China: Social App Capture-Recapture Method. JMIR. Mhealth. Uhealth..

[b0100] Hu Y., Zhong X.N., Peng B., Zhang Y., Liang H. (2019). Comparison of depression and anxiety between HIV-negative men who have sex with men and women (MSMW) and men who have sex with men only (MSMO): a cross-sectional study in Western China. BMJ Open..

[b0105] Ito H., Yamamoto T., Morita S. (2021). The effect of men who have sex with men (MSM) on the spread of sexually transmitted infections. Theor. Bio.l Med. Model..

[b0110] Jeffries W.L. (4th., 2014.). Beyond the bisexual bridge: sexual health among U.S. men who have sex with men and women. Am. J. Prev. Med..

[b0115] Jeffries W.L., Johnson O.D. (2018). Internalized Homonegativity and Substance Use Among U.S. Men who have sex with men only (MSMO) and men who have sex with men and women (MSMW). Subst. Use. Misuse..

[b0120] Jin M., Yang Z., Dong Z., Han J. (2013). Correlates of consistent condom use among men who have sex with men recruited through the Internet in Huzhou city: a cross-sectional survey. BMC Public Health..

[b0125] Kirwan M., Stewart R., Chen W., Hammett J.F., Davis K.C. (2023). Sexual Compulsivity Mediates the Association Between Childhood Sexual Abuse and Condom Use Resistance Among Men Who Have Sex with Men and Women. Arch. Sex. Behav..

[b0130] Kloek M., Bulstra C.A., van Noord L., Al-Hassany L., Cowan F.M., Hontelez J.A.C. (2022). HIV prevalence among men who have sex with men, transgender women and cisgender male sex workers in sub-Saharan Africa: a systematic review and meta-analysis. J. Int. AIDS. Soc..

[b0135] Mann L.M., Le G.A., Goodreau S.M. (2022). Correlations between community-level HIV preexposure prophylaxis coverage and individual-level sexual behaviors among United States MSM. AIDS..

[b0140] Martín-Sánchez M., Case R., Fairley C. (2020). Trends and differences in sexual practices and sexually transmitted infections in men who have sex with men only (MSMO) and men who have sex with men and women (MSMW): a repeated cross-sectional study in Melbourne. Australia. BMJ Open..

[b0145] Mc C.D.H., Oster A.M., Jeffries W.L. (2017). HIV acquisition and transmission among men who have sex with men and women: What we know and how to prevent it. Prev. Med..

[b0150] Miltz A.R., Lampe F.C., Bacchus L.J. (2019). Intimate partner violence, depression, and sexual behaviour among gay, bisexual and other men who have sex with men in the PROUD trial. BMC Public Health..

[b0155] Mimiaga M.J., Pantalone D.W., Biello K.B. (2018). A randomized controlled efficacy trial of behavioral activation for concurrent stimulant use and sexual risk for HIV acquisition among MSM: project IMPACT study protocol. BMC Public Health..

[b0160] Nyoni P., James N. (2022). Condom use and risk factors of inconsistent or low use of the condoms during heterosexual anal intercourse in sub-Saharan Africa: a scoping review. Afr. Health. Sci..

[b0165] Olawore O., Crowell T.A., Ketende S.C. (2021). Individual and partnership characteristics associated with consistent condom use in a cohort of cisgender men who have sex with men and transgender women in Nigeria. BMC Public Health..

[b0170] O'Leary, A., Jones, KT., 2006. Bisexual men and heterosexual women: how big is the bridge? How can we know? Sex. Transm. Dis. Oct;33(10):594-5. doi: 10.1097/01.olq.0000225280.44538.f6. 10.1097/01.olq.0000225280.44538.f6 16837828

[b0175] Operario D., Sun S., Bermudez A.N. (2022). Integrating HIV and mental health interventions to address a global syndemic among men who have sex with men. Lancet HIV..

[b0180] Parchem B., Aguayo-Romero R.A., Alizaga N.M., Del Río-González A.M., Poppen P.J., Zea M.C. (2022). Identity and Relational Factors Associated with Sexual Role and Positioning for Anal Sex among Colombian Sexual Minority Men. J. Sex. Res..

[b0185] Pravosud V., Ballard A.M., Holloway I.W., Young A.M. (2022). Online Partner Seeking and Sexual Behaviors Among Men Who Have Sex With Men From Small and Midsized Towns: Cross-sectional Study. JMIR. Form. Res..

[b0190] Ramakrishnan L., Ramanathan S., Chakrapani V. (2015). Comparison of Sexual Risk, HIV/STI Prevalence and Intervention Exposure Among Men Who Have Sex with Men and Women (MSMW) and Men Who Have Sex with Men Only (MSMO) in India: Implications for HIV Prevention. AIDS. Behav..

[b0195] Rosińska M., Gios L., Nöstlinger C. (2018). Prevalence of drug use during sex amongst MSM in Europe: Results from a multi-site bio-behavioural survey. Int. J. Drug Policy..

[b0200] Shannon C.L., Klausner J.D. (2018). The growing epidemic of sexually transmitted infections in adolescents: a neglected population. Curr. Opin. Pediatr..

[b0205] Stansfield S.E., Mittler J.E., Gottlieb G.S. (2019). Sexual role and HIV-1 set point viral load among men who have sex with men. Epidemics..

[b0210] Unaids The path that ends AIDS: UNAIDS Global AIDS Update 2023. Geneva: Joint United Nations Programme on HIV/AIDS 2023.

[b0215] Zhao H., Liu H., Wang L. (2020). Epidemiological Characteristics of Newly-Reported HIV Cases Among Youth Aged 15–24 Years - China, 2010–2019. China CDC Wkly..

[b0220] Zhou H., Zhu Y.Y., Gao Y.Y. (2023). Online distribution of HIV self-testing kits to promote HIV testing among men who have sex with men discontinuing pre-exposure prophylaxis after demonstration project completion in China: a multicentre open-label randomized controlled trial. Lancet. Reg. Health. West. Pac..

